# How to evaluate digital leadership: a cross-sectional study

**DOI:** 10.1186/s12995-021-00335-x

**Published:** 2021-10-01

**Authors:** Kevin Claassen, Dominique Rodil dos Anjos, Jan Kettschau, Horst Christoph Broding

**Affiliations:** Chair of Occupational Medicine and Corporate Health Management, Faculty of Health, Department of Human Medicine, Witten/Herdecke University, Alfred-Herrhausen-Straße 50, 58448 Witten, Germany

**Keywords:** Digitalization, Digital leadership, Index, Score, Municipal administration, Digitalisierung, digitale Führung, Index, Score, Stadtverwaltung

## Abstract

**Background:**

With the increasing digitalization of the working environment, the demands on managers are changing fundamentally to the point of an emerging field of research in digital leadership. Municipal administrations are particularly affected by the digital transformation processes. Therefore, a score to measure the construct of digital leadership competence in the context of virtual-based workstation was developed and tested.

**Methods:**

Based on an online survey with *n* = 546 employees at virtual-based workstations in municipal administrations in 2020, the instrument is tested regarding selectivity (coefficients), dimensionality (principal component analysis), homogeneity (inter-product-moment correlations), reliability (Cronbach’s α) and construct validity (correlation with general leadership skills).

**Results:**

The instrument can be considered selective, one-dimensional, homogeneous, reliable and constructively valid in the sense of the formulated hypotheses. By integrating the employees’ perspective, the instrument aims to be one of the first of its kind to initiate a scientific further discourse. Among other things, the categorization of the co-determination component as either traditional or digital leadership can be discussed.

**Conclusions:**

The developed instrument for measuring digital leadership performs well concerning the aspects of discriminatory power, one-dimensionality, homogeneity, reliability as well as construct validity. It aims to induce further research and a scientific discourse on the topic of health-oriented leadership within the world of work 4.0.

## Abstract (de)

**Hintergrund:**

Mit der zunehmenden Digitalisierung der Arbeitswelt verändern sich die Anforderungen an Führungskräfte dergestalt grundlegend, dass daraus das Forschungsfeld der digitalen Führung entsteht. Kommunale Stadtverwaltungen sind dabei in besonderem Maße von digitalen Transformationsprozessen betroffen, sodass in diesem Kontext ein Score zur Messung des Konstrukts digitale Führungskompetenz am Bildschirmarbeitsplatz entwickelt und getestet wurde.

**Methoden:**

Auf Grundlage einer Online-Befragung von *n* = 546 Beschäftigten an Bildschirmarbeitsplätzen in Stadtverwaltungen aus dem Jahr 2020 wird das Instrument hinsichtlich Trennschärfe(-koeffizienten), Dimensionalität (Hauptkomponentenanalyse), Homogenität (Inter-Produkt-Moment-Korrelationen), Reliabilität (Cronbachs α) und Konstruktvalidität (Korrelation mit allgemeiner Führungskompetenz) geprüft.

**Ergebnisse:**

Das Instrument kann als trennscharf, eindimensional, homogen, reliabel und konstruktvalide im Sinne der aufgestellten Hypothesen betrachtet werden. Durch die Integration der Beschäftigtenperspektive zielt das Instrument als eines der ersten dieser Art auf die Initiierung eines wissenschaftlichen Diskurses. Uneindeutig zeigt sich unter anderem die Zuordnung der Komponente Mitbestimmung im Spannungsfeld zwischen klassischer und digitaler Führung.

**Schlussfolgerung:**

Das entwickelte Instrument zur Messung digitaler Führung schneidet hinsichtlich der Aspekte Trennschärfe, Eindimensionalität, Homogenität, Reliabilität sowie Konstruktvalidität gut ab. Es soll weitere Forschung und einen wissenschaftlichen Diskurs zum Thema gesundheitsorientierte Führung in der Arbeitswelt 4.0 anregen.

## Background

This article presents an instrument for measuring the construct of digital leadership competence at the computer workstation. Digitalization is considered one of the mega-trends that is causing profound social change in the sense of a transformation of living and working environments [1].

Traum et al. (2017), as part of the KODIMA project, developed a definition that explicitly includes the working individual affected by digitalization:

“Digitalization is the introduction or increased use of information and communication technologies (ICT) by (working) individuals, organizations, economic sectors and societies with the characteristic consequences of acceleration, increasing abstractness, flexibilization, and individualization of processes and outcomes.” [2]

Piasecki (2020) draws a narrower frame of reference around municipal administrations and describes digitalization as “essentially the shift of administrative tasks to a new digital level and the integration of traditional (paper-based) processes into computer-based processing structures to optimize results and accelerate procedures” [3]. The goal of work-related digitalization is the transition to the world of work 4.0, in which routine activities are replaced by knowledge-based decision-making with complex, dynamically changing activities. Thus, office work can be organized and designed more individually. The expansion of existing technologies encourages mobile working at flexible workplaces with flexible working hours [4]. Work 4.0, has established itself as a signal term referring to the fundamental structural change in gainful employment resulting from advancing digitalization [5]. Digital and mobile communication enable companies to collaborate and coordinate over greater spatial distance as well as with temporal flexibility. It also facilitates access to specialized knowledge, expertise, and resources [6]. A variety of new work models result from the changing work opportunities. Boundaries in different areas, such as between locations, companies, customers, and workforces are becoming increasingly blurred [6]. Routine activities become more and more automated, so much that tasks for employees can be designed to be more cross-functional and cross-divisional. Their work becomes increasingly information-based. The targeted further qualification of the workforce is of crucial importance [6].

Consequently, also the demands on managers are subject to digital transformation processes, which is reflected in society’s understanding of leadership. The concept of employee leadership and the demands placed on managers keep changing as digitalization progresses.

In general, leadership is required whenever several people work on problem solutions in a division of labor with need for coordination [7, 8]. “The increasing complexity of organizations and work requires leaders to manage people as effectively and efficiently as possible” [8]. Socially, the notion of leadership is evolving since the beginning of the twentieth century from unidirectional control to a holistic, reciprocal influence in which leaders solicit and use employee feedback for advancement [9, 10].

Targeting digital leadership culture, Meier et al. (2017) extracted four key characteristics from the interactive leadership approach: collaborative, integer and social, inspiring and open, fostering resilience [11].

As in literature the term digital leadership is not defined consistently, different emphases are placed on it. Promsri (2019) compiles 64 characteristics of digital leadership in a review paper and aggregates them into six key characteristics of a digital leader [12]:

1. Digital knowledge and literacy - knowledge of the possibilities of digitalization-related changes;
2. Vision - clear objective regarding desired digital transformation processes;
3. Customer focus - taking into account the expectations and wishes of customers with regard to digital processes;
4. Agility - good adaptability toward the rapidly changing work processes;
5. Risk-taking (creation of an experimental atmosphere) - establishing a culture of constructive criticism that enables trial and error as well as innovation;
6. Collaboration - strengthening the cooperation among employees in terms of location, time, culture, etc.

Overall, there is an observable trend from rigid, hierarchical management toward dynamic decision-making processes with flat hierarchies, joint decision-making and changing responsibilities. Social skills become increasingly relevant alongside expert knowledge [6, 11, 12]. This trend is expressed in the empowerment approach [13]. Individual, employee-related empowerment aims to influence the perception of the employee role positively. Accordingly, the perception of one’s own significance, competence, self-determination, and influence during work should be strengthened [13]. Central connections to successful digital work can be found in the experienced self-determination and the experienced influence on the working process. This goes hand in hand with greater freedom of choice for employees regarding working hours, work location and the sequence of working processes. Flattened hierarchies in project groups also enable and require self-organization with changing leadership role focused on personal skills [4, 6].

The results of current reviews [14, 15] indicate that positive leadership styles and behaviors are associated with better health, less health complaints and less stress experience. Negative leadership behaviors as a risk factor are analyzed significantly less. Nevertheless, the reviews point out that negative leadership behavior is associated with low psychological well-being, lower job satisfaction and higher sick leave [15]. The “health-oriented leadership” (HoL) approach of Franke and Felfe goes beyond these studies and provides a broader model of health-specific leadership behavior. Within this approach, more aspects of a leader’s communication and the health-promoting design of working conditions are integrated. In addition, values and awareness of managers towards the health of their employees as well as the awareness and behavior of the employees themselves are addressed [16].

From the transformation processes described above, a need for evaluating digital leadership styles can be derived. A particular need arises in Germany around municipal administrations. This is due to the fact that they are, for example, obligated to keep digital records and offer electronic citizen services according to the framework of the Act to Promote Electronic Administration in North Rhine-Westphalia. While it is also called E-Government Act and the municipal administrations have accepted the challenge, there is a need to operationalize digital leadership.

Existing approaches for constructing an index of digital leadership competence refer to small and medium-sized enterprises [17], are based on a survey of the executives themselves [18], or do not have a sufficient number of cases for validation [19]. Other existing approaches are used for personnel selection and classification of managers [20, 21].

The score proposed in the following, on the other hand, is based on the subjective perspective of managed employees at VDU workstations in municipal administrations and was developed as part of the project “Health and Digital Change” (GudW), in which it is also being tested. The score is called “DigiFuehr” due to the German word “Führung”, which means leadership. The following hypotheses are to be tested:

1. The items of the DigiFuehr score have a high discriminatory power, i.e. all items have at least medium correlations with the remaining overall construct (*r* > .3).
2. The DigiFuehr score measures a one-dimensional construct, i.e., in a principal component analysis only one factor can be extracted that has an eigenvalue greater than one (EV > 1).
3. The items of the DigiFuehr score are homogeneous, i.e. they show at least medium correlations among each other (*r* > .3).
4. The items of the DigiFuehr score are highly reliable, i.e. they show a high internal consistency (α > .8).
5. The DigiFuehr score can be construct-validated via an analogous summative score to classic leadership (called “ClassicFuehr”), i.e., the two scores have at least a medium correlation with each other (*r* > .3).

## Methods

Following a literature review and joint consultations with eight experts from the human resources (HR) and occupational health management (OHM) fields in the steering committee of the GudW project, seven core areas of digital leadership competence were identified. These core areas are operationalized by the closed questions listed in Table 1. This results in the following four-stage response options with point values in parentheses: not applicable (1), rather not applicable (2), rather applicable (3), fully applicable (4). These seven items are summed up, resulting in a point score in the sense of a Likert scale [22], which is referred to as “DigiFuehr”. Finally, the values are projected onto the value range between 0 and 100 for the sake of clarity. The addressees of the survey are digitally managed employees.

**Table 1:** Items of the summative score DigiFuehr

Description	Formulation
DigiFuehr 1	*“I am involved in decisions that affect my work and my digital work environment.”*
DigiFuehr 2	*“My digital literacy is encouraged by my manager.”*
DigiFuehr 3	*“When there is a need for questions about* digitalization*, I receive support from my manager.”*
DigiFuehr 4	*“I get regular feedback on the quality of my digital work.”*
DigiFuehr 5	*“I get all the information I need to do my digital job.”*
DigiFuehr 6	*“I am supported by my manager to better understand and use digital applications.”*
DigiFuehr 7	*“In my department, digital working methods are encouraged.”*

The score was tested in an online survey in the municipal administrations of three model regions in North Rhine-Westphalia participating in the GudW project. The model regions were selected in such a way that the entire federal state was sufficiently represented structurally, for example in terms of the relationship between urban and rural regions and sociocultural dimensions. Inclusion criterion was a (pre-pandemic) presence activity at the VDU workplace. In this way, 1319 employees were invited to participate in an online survey in October 2020, of whom 710 had taken part in the survey up to and including December 2020 (response rate: 53.83%), after a reminder was sent out again in November. Ex post, employees with own management responsibilities were excluded, resulting in a net case number of *n* = 546 employees, whose sociodemographic are listed in Table 2. Since item nonresponse was found to be between 24 and 34%, depending on the item, the missing values were filled using multiple imputation (MICE) following Rubin (1987) [23]. As the proportion of missing values related to leadership roughly equals the proportion of missing values related to the socio-demographic variables described below, we assumed missing at random (MAR) and used all leadership as well as demographic variables as predictors applying predictive mean matching.

**Table 2:** Sociodemographic of the respondents in the project (*n* = 546)

Gender: female	63,68%
Age in years	43,57 (± 12,95)
Education: At least university entrance qualification	64,71%
Model region I	30,95%
Model region II	31,32%
Model region III	37,73%

Alternatively, in order to test the influence of the items on symmetry and thus the legitimacy of summation, a score is constructed based on the contribution of the individual items to the overall correlation (product-moment correlation). In addition, a normal distribution is tested using the Shapiro-Wilk test [24].

The first hypothesis is tested using coefficients of discriminatory power, for which a part-whole correction was applied [25]. The classification of the strength of the association is based on Cohen [26]. For the second hypothesis test, a principal component analysis (PCA) is calculated using eigenvalue decomposition of the standardized correlation matrix [27]. The homogeneity of the score (hypothesis three) is assessed using the intercorrelation matrix itself, and the internal consistency of the score (hypothesis four) is assessed using Cronbach’s α [28].

A test of construct validity formulated in hypothesis five is conducted via a correlation analysis with a score of classical leadership competence. The score is called “ClassicFuehr” and was constructed with the items listed in Table 3, based on the same survey. This method is completely analogous to the construction of the DigiFuehr score, however focusing on leadership traits, which are consistent with an analogous working world. Imperfect reliability of the two constructs is accounted for with a double reduction correction [29].

**Table 3:** Items of the summative score ClassicFuehr

Description	Formulation
ClassicFuehr 1	*“My professional development is encouraged.”*
ClassicFuehr 2	*“I am supported in balancing my work and personal life.”*
ClassicFuehr 3	*“I receive recognition and appreciation for my work.”*
ClassicFuehr 4	*“I feel treated fairly, decisions are transparent and understandable.”*
ClassicFuehr 5	*“I feel like I’m allowed to make mistakes.”*
ClassicFuehr 6	*“Phases where I have more stress or less stress are balanced.”*
ClassicFuehr 7	*“I know exactly what is expected from me.”*

## Results

An alternative correlation-based score leads to the weighting of the items between 11 and 16% mentioned in Table 4, so that symmetry can be assumed approximately and a summation also seems justified for the sake of simplicity.

**Table 4:** Weighting of the correlative score formation

Description	Weighting
DigiFuehr 1	11%
DigiFuehr 2	16%
DigiFuehr 3	16%
DigiFuehr 4	14%
DigiFuehr 5	13%
DigiFuehr 6	16%
DigiFuehr 7	14%

**Fig. 1:**
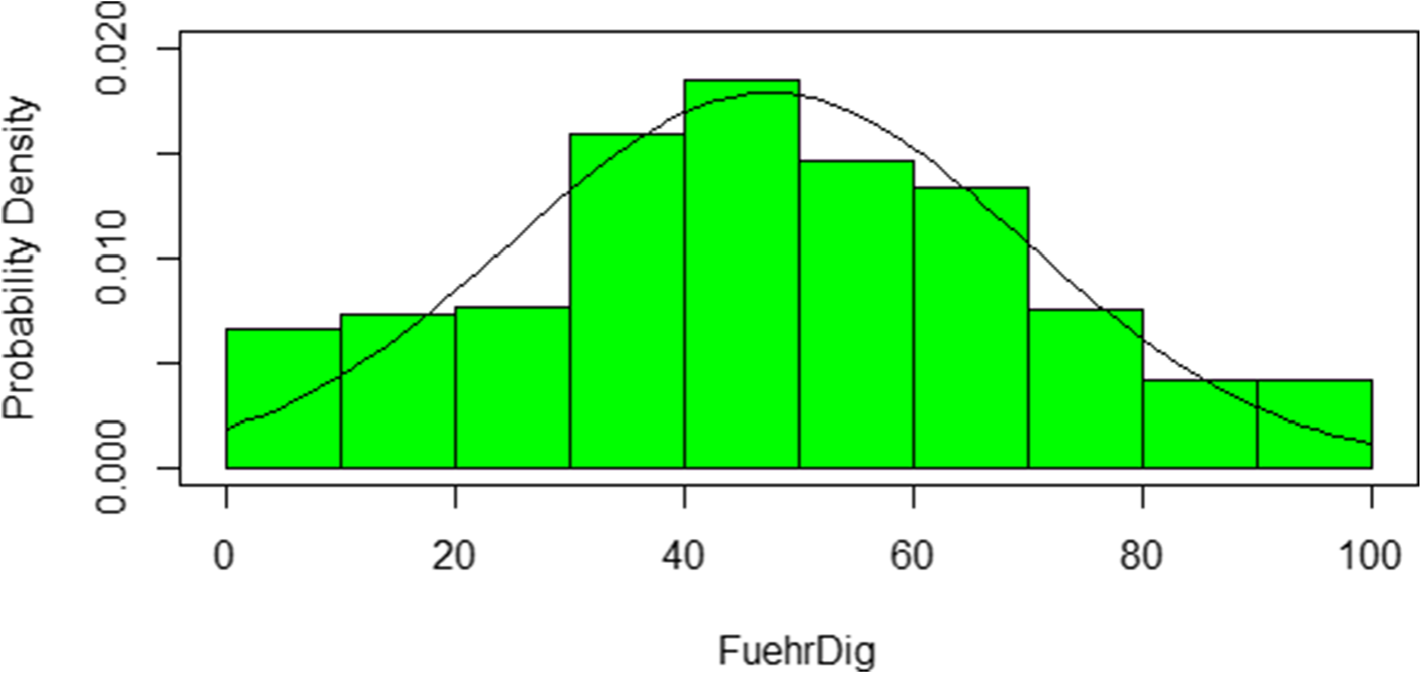
Plot of the probability density

Table 5 shows the means and standard deviations of the initial variables and of the score transformed to the range of values between 0 and 100. A plot of the probability density is provided in Fig. 1, which shows approximately a normal distribution in terms of the plotted bell curve, which can be confirmed computationally using Shapiro-Wilk (*p* < .001).

**Table 5:** Means, standard deviations (*n* = 546)

Description	Arithmetic mean (***±*** standard deviation)
DigiFuehr 1	2,36 (± 0,86)
DigiFuehr 2	2,32 (± 0,87)
DigiFuehr 3	2,52 (± 0,92)
DigiFuehr 4	2,14 (± 0,88)
DigiFuehr 5	2,59 (± 0,80)
DigiFuehr 6	2,30 (± 0,94)
DigiFuehr 7	2,69 (± 0,88)
DigiFuehr	47,33 (± 22,26)

Table 6 points to an acceptable discriminatory power of the items, because all correlations are above .4. While co-determination (DigiFuehr_SQ001) is the least related to the remaining overall construct (*r* = .47). This can also be seen from the inter-correlation matrix. Cronbach’s α = .88 indicates high internal consistency and thus, as a surrogate parameter, high reliability of the score.

**Table 6:** Inter-correlation matrix (DigiFuehr describes the part-whole correlations) and Cronbach’s α excl. DigiFuehr (*n* = 546)

α = 0,88	DigiFuehr 1	DigiFuehr 2	DigiFuehr 3	DigiFuehr 4	DigiFuehr 5	DigiFuehr 6	DigiFuehr 7	DigiFuehr
DigiFuehr 1	1	0,52	0,34	0,36	0,39	0,34	0,31	0,47
DigiFuehr 2	0,52	1	0,66	0,57	0,47	0,70	0,52	0,76
DigiFuehr 3	0,34	0,66	1	0,55	0,52	0,77	0,57	0,75
DigiFuehr 4	0,36	0,57	0,55	1	0,51	0,60	0,49	0,67
DigiFuehr 5	0,39	0,47	0,52	0,51	1	0,51	0,43	0,61
DigiFuehr 6	0,34	0,70	0,77	0,60	0,51	1	0,57	0,77
DigiFuehr 7	0,31	0,52	0,57	0,49	0,43	0,57	1	0,62
DigiFuehr	0,47	0,76	0,75	0,67	0,61	0,77	0,62	1

**Fig. 2:**
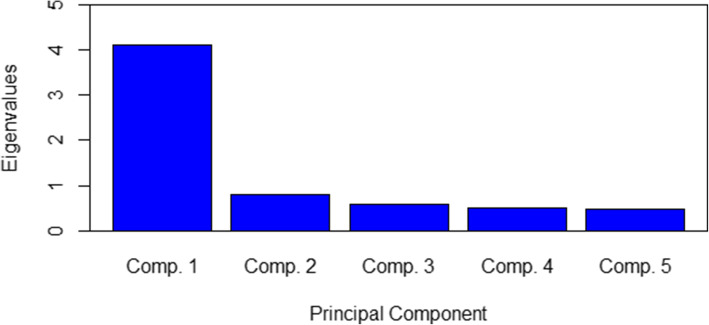
Scree plot eigenvalues

By means of principal component analysis, one factor could be extracted that explained 58.18% of the total variance of the items and obtained an eigenvalue of 4.06, respectively, explaining as much as 4.06 manifest variables. The eigenvalues of the other extracted factors are below one, which is displayed graphically in Fig. 2 in as a scree plot. However, it is striking that the second factor extracted is highly correlated with Co-Determination (DigiFuehr1, *r* = 0.77) while every other correlation of an item to this factor is below .2.

While the analogous score measuring classical leadership competence shows an acceptable reliability itself (α = .81), it is moderately correlated with DigiFuehr (*r* = .42) after a double reduction correction was performed in order to adjust for the imperfect reliability of both constructs. Thus, the previously established hypotheses one to five can be confirmed.

## Discussion

The DigiFuehr score was validated as an employee-oriented instrument for measuring digital leadership competence in municipal administrations in the sense that its items are selective, homogeneous and reliable. The score shows a moderate correlation with classic leadership competencies, indicating that additional competencies are required with regard to Work 4.0.

While the summative instrument in its current form is indeed selective, homogenous and (rather) one-dimensional, the item targeting co-determination deviates from the other components due to a noticeable overlap with an independent second factor that could be extracted from the data using PCA. This is expectable when considering that co-determination depends on autonomy and participation while the remaining items are mainly support- and process-oriented. As a requirement of autonomy, it is crucial how far a digital leader is willing to enable cooperation and self-organization within his team. On this account, further research is well advised to investigate whether co-determination should be modelled as an independent component of digital leadership. This can be achieved via a confirmatory factor analysis when more questions on co-determination and autonomy are included. Until then, the usage of DigiFuehr is recommended in its current form (instead of skipping the question on co-determination) as many theories of digital leadership focus on the reciprocal dimension of leadership, which underlines the general relevance of co-determination for digital leadership.

What should also be emphasized is that in the present study, as experts of themselves, employees without management responsibility (in municipal administration) were deliberately surveyed, whose needs may well collide with the interests of managers as well as of the top management. However, the paradigm shift in leadership described above also explicitly includes a resource-oriented empowerment approach. Thus, it is suggested that following further surveys examine the extent to which the assessments of managers and those managed (about digital leadership competence) converge. The extent to which such an approach may have already been internalized would be valuable to analyze.

Because the current score was validated in the setting of the municipal administration, further research is also necessary to evaluate its validity in measuring digital leadership competencies in other VDU workplaces. In this context, it should be considered that only employees were included who already worked at the VDU workplace before the Covid-19 pandemic. This could lead to a reduction of external validity, as less experienced employees first introduced to a digitalized work environment are not represented.

Moreover, classical leadership was measured via a non-standardized instrument (ClassicFuehr), because some of its items were not only based on existing literature but also influenced by the results of a group discussion with project partners as representatives of the digital model regions. Hence, the construct could suffer from reduced internal validity.

Consequently and because it is not exhaustive with regard to the topic of leadership at a distance, the score has the character of a suggestion that is intended to initiate a scientific discourse, whereby there is a fundamental openness to modifications and improvements.

## Conclusions

In recent years, there has been a particular increase in scientific interest in the relationship between leadership and employee health. Meanwhile, the digitalization of work processes advances, affecting the management regarding VDU workplaces in municipal administrations. Therefore, a standardized instrument for measuring digital leadership was developed and tested. It performs well concerning the aspects of discriminatory power, one-dimensionality, homogeneity, reliability as well as construct validity. It aims to induce further research and a scientific discourse on the topic of health-oriented leadership within the world of work 4.0.

## Data Availability

The dataset supporting the conclusions of this article is included within the article. Data available on reasonable request.

## Abbreviations

EV: Eigenvalue
GudW: Health and Digital change (German: Gesundheit und digitaler Wandel)
HoL: Health-oriented leadership
HR: Human resources
OHM: Occupational health management
PCA: Principal component analysis
VDU: Visual display unit
